# Anticancer Potential of Raddeanin A, a Natural Triterpenoid Isolated from *Anemone raddeana Regel*

**DOI:** 10.3390/molecules25051035

**Published:** 2020-02-25

**Authors:** Irum Naz, Shanaya Ramchandani, Muhammad Rashid Khan, Min Hee Yang, Kwang Seok Ahn

**Affiliations:** 1 Department of Biochemistry, Faculty of Biological Sciences, Quaid-i-Azam University, Islamabad 45320, Pakistan; irumnaz@ps.qau.edu.pk; 2 The University of Melbourne, Parkville, VIC 3010, Australia; ramchandanishanaya@gmail.com; 3 Higher Education Commission of Pakistan, Islamabad 44000, Pakistan; 4 KHU-KIST Department of Converging Science and Technology, Kyung Hee University, Seoul 02447, Korea; didmini@naver.com

**Keywords:** raddeanin A, malignant, PI3K/AKT, signaling pathways

## Abstract

Natural compounds extracted from plants have gained immense importance in the fight against cancer cells due to their lesser toxicity and potential therapeutic effects. Raddeanin A (RA), an oleanane type triterpenoid is a major compound isolated from *Anemone raddeana Regel*. As an anticancer agent, RA induces apoptosis, cell cycle arrest, inhibits invasion, migration and angiogenesis in malignant cell lines as well as in preclinical models. In this systemic review, the pharmacological effects of RA and its underlying molecular mechanisms were carefully analyzed and potential molecular targets have been highlighted. The apoptotic potential of RA can be mediated through the modulation of Bcl-2, Bax, caspase-3, caspase-8, caspase-9, cytochrome c and poly-ADP ribose polymerase (PARP) cleavage. PI3K/Akt signaling pathway serves as the major molecular target affected by RA. Furthermore, RA can block cell proliferation through inhibition of canonical Wnt/β-catenin signaling pathway in colorectal cancer cells. RA can also alter the activation of NF-κB and STAT3 signaling pathways to suppress invasion and metastasis. RA has also exhibited promising anticancer potential against drug resistant cancer cells and can enhance the anticancer effects of several chemotherapeutic agents. Overall, RA may function as a promising compound in combating cancer, although further in-depth study is required under clinical settings to validate its efficacy in cancer patients.

## 1. Introduction

Cancer is a disease which arises through uncontrolled cell division leading to the formation of a tumor, which metastasizes to other body parts through the lymphatic and circulatory systems [1]. According to latest reports, around 18.1 million people were affected worldwide from different types of cancer in 2018. Up to 9.1 million cancer deaths were reported in 2018 and an increase up to 20.3 million is expected in 2026 [2]. Asia had the highest cancer death rate (57.3%), followed by Europe and America at 20.3% and 14.4%, respectively. Most of the deaths were attributed to lung cancer (2.1 million) followed by breast cancer (2.1 million) and colorectal cancer (1.8 million) [3]. The mortality rate of cancer can be reduced by understanding the etiology of cancer through advancement of diagnosis techniques, prevention strategies and treatment [4]. Cancer therapies including surgery, radiotherapy, chemotherapy and molecular targeted therapy have stabilized cancer prevalence to some extent [5], but unfortunately, these therapeutic strategies have been found to be less effective due to late diagnosis, lack of selective therapeutics, high toxicity, and ability of cancer cells to develop resistance against available treatments [6]. Therefore, several attempts have been made to develop novel treatments in fighting cancer and to improve patient survival.

Along with various available treatments, the use of natural plant products as medicines provides a new horizon for the treatment of various types of diseases [7,8,9,10,11]. The source of about 60% of the available drugs are natural raw materials from plants [12,13,14,15,16,17]. Natural drugs being used in clinics such as paclitaxel, vinblastine and camptothecin have gained a considerable amount of importance due to their reduced side effects [18]. Saponins, particularly triterpenoid saponins, have been used in Traditional Chinese Medicine (TCM) to cure cancer malignancies, for instance, they target the metastatic, invasive and angiogenic potential of cancer cells [19,20,21]. Saponins are also reported to reduce the resistance of neoplastic cells against chemotherapeutic agents, which is one of the protective mechanisms of cancer cells [22,23]. The progression of cancer cells is primarily mediated through the activation of multiple signaling pathways including the deregulation of MAPK, JAK/STAT and PI3K/Akt pathways which support carcinogenesis [24,25,26,27].

*Anemone raddeana* Regel is a medicinal plant, recognized by its vernacular name as “Liangtoujian” in China. The distribution of this plant is not only limited to China, but throughout the globe, particularly in Russia, Korea, and Japan [28,29]. The rhizome of *Anemone raddeana* Regel is used in Chinese conventional therapies to cure rheumatism, arthritis, neuralgia, paralysis and other diseases [30,31,32]. The anemone herb contains oleanane triterpenoid saponins accompanied by lactones, alkaloids, saccharide, triterpenoids and fats [33,34]. In the past few years, various bioactive saponins compounds have been extracted from this plant, including RA, which has gained importance due to its anti-inflammatory, analgesic, and antitumor activities [33]. 

**Figure 1:**
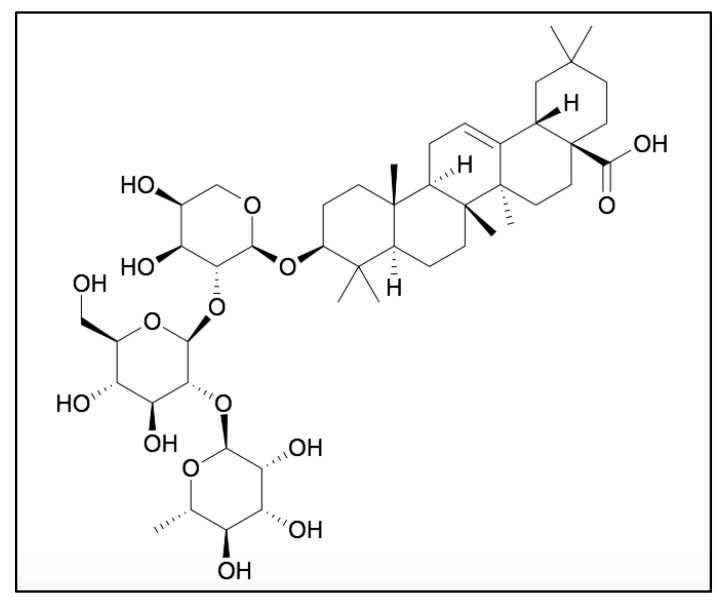
Molecular structure of RA.

RA (C47H76O10) is an oleanane class triterpenoid saponin, isolated from the roots of *Anemone raddeana*, possessing a 3-O-α-L-rhamnopyranosyl-(1→2)-β-D-glucopyranosyl-(1→2)-α-L- arabino- pyranoside as the sugar moiety (Figure 1) [31]. The anticancer potential of RA is linked with the presence of sugar moieties and its carboxyl group (-COOH) at the C-28 position (Figure 1). The free carboxyl group plays an important role in the cytotoxic potential of RA. In addition, the presence of rhamnopyranose, glucopyranose and arabinopyranosyl groups can also act as important moieties to enhance its therapeutic potential. 

A study reported that the modification at C-28 through esterification can lead to the reduction of cytotoxic potential, thus further confirming the role of this carbon moiety in the biological activities reported for RA [31]. Various studies has shown that RA possesses cytotoxic potential through inhibition of proliferation, invasion and induction of apoptosis in multiple human carcinogenic cells including breast cancer, hepatocellular carcinoma, gastric cancer, and non-small cell lung carcinoma cells [35,36,37,38]. In addition, RA has also shown inhibitory properties at low concentrations against histone deacetylases (HDACs), further suggesting its cytotoxicity against cancer cells [31]. Likewise, on account of its reported pharmacological safety, RA can be used in combination with other anti-cancer drugs to enhance the sensitivity against resistant tumor cells. In this review article, the cytotoxic and therapeutic potential of RA has been comprehensively analyzed. 

## 2. Molecular Targets of RA

**Figure 2:**
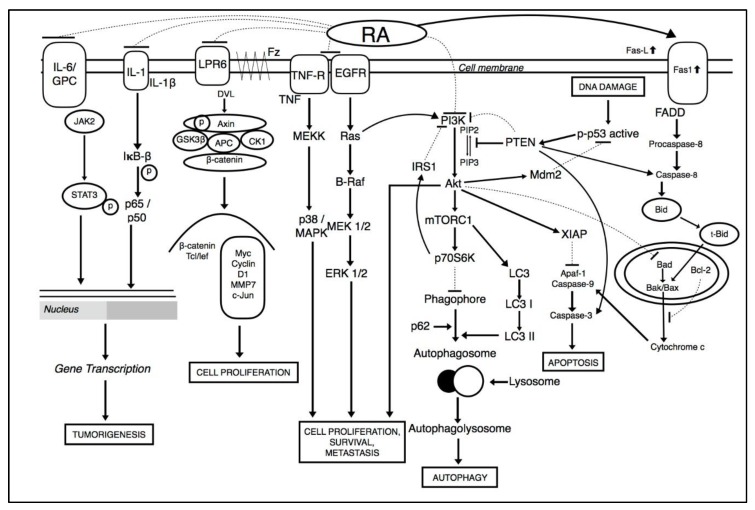
Multiple molecular pathways affected by RA.

Tumorigenic cells can lead to the deregulation of multiple signaling pathways. Hence, compounds that act on multiple targets are required in the deterrence and treatment of cancer malignancies [39,40,41,42]. Various studies show the consecutive activation of major signaling pathways which leads to activation of downstream proteins, and contributes to survival, angiogenesis, and metastasis of cancer cells (illustrated in Figure 2). These include the PI3K/Akt pathway, signal transducer and activator of transcription 3 (STAT3) pathway, and nuclear-factor kappa B (NF-κB) pathway [43,44,45,46,47,48,49,50,51,52,53,54]. 

Among these pathways, PI3K/Akt pathway is a major signaling pathway that is constitutively turned on and can activate a number of downstream proteins during carcinogenesis. PI3K/Akt is a serine-threonine kinase which is regulated by phosphoinositide dependent kinase-1 (PDK1) when it gets bound with phosphatidylinositol trisphosphate (PIP_3_) on the surface of cell membrane [44,55,56,57,58,59,60,61]. PDK1 phosphorylates AKT to p-AKT which indirectly activates mTOR pathway [44,55,56,57]. Together, PI3K/AKT/mTOR signaling pathway can regulate cellular proliferation, metabolism, survival, and angiogenesis [62,63,64,65,66]. RA treatment reduced cancer cell proliferation by modulating the PI3K/Akt signaling pathway [67]. RA was found to reduced angiogenesis and autophagy by abrogating Akt/PI3K mediated activation of mTOR pathway in breast cancer cells. Together, PI3K/Akt/mTOR lead to the activation of EeF-2K which is a key regulator of autophagy [66,67,68]. In addition, RA also induces apoptosis in multiple cancer through the downregulation of mitochondrial dependent pathways. As potent inducer, it inhibits pro-survival of B-cell lymphoma 2 family members (Bcl-2) and induces the activation of Bax (Bcl-2 associated X protein), cytochrome c, activated caspase-3, caspase-8, caspase-9 and poly-ADP ribose polymerase (PARP) cleavage. RA also downregulated cell proliferation through the inhibition of cyclins and cyclin dependent kinase complexes such as cyclin E/CDK2 and cyclin D1/CDK4 [69]. Moreover, RA effectively inhibited NF-κB ligand (RANKL) which mediated the upregulation of downstream signaling pathways such as NF-κB, MAPK, and SRC/AKT, to affect osteoclast differentiation, which results in bone resorption in breast cancer [69,70,71,72].

RA treatment effectively inhibited the initiation and proliferation of colorectal cancer cells through the suppression of the canonical Wnt/β-catenin signaling pathway. The inhibition was conducted by inactivating the Wnt co-receptor LRP6 and phosphorylating GSK-3β, an activator of downstream target genes c-Myc and Cyclin D1 [45,73,74]. c-Myc activation links with the activation of pleiotropic transcription factor, cell cycle progression, proliferation, and metabolism. Furthermore, treatment with RA suppressed invasion and osteosarcomas through the modulation of NF-κB signaling pathways by targeting IκBα phosphorylation to attenuate transcriptional activity of the NF-κB signaling pathway. These mechanisms lead to the downregulation of downstream matrix metallopeptidase-2 (MMP-2) and matrix metallopeptidase-9 (MMP-9) proteins [37,52]. These proteins are associated with the invasive and migratory potential of cancer cells [37]. Another finding reveals that RA initiated apoptosis and inhibited metastasis in osteosarcoma cells by stimulating ROS levels to trigger JNK activation. RA also suppressed MAPK and ERK pathways involved in resistance of cell growth, metabolism, autophagy [75], induction of apoptosis [76,77], and invasion [37]. In addition, RA has been reported to modulate STAT3 transcription factors involved in the expression of a large number of genes which plays a role in many physiological processes such as development, differentiation, metabolism, immunity and cancer progression in osteosarcoma [78]. RA was found as a potential antiangiogenic candidate through the modulation of VEGF mediated phosphorylation of VEGFR2 and downstream protein kinases FAK, PLCγ1, JAK2, Src and Akt in colorectal cancer tumors [79]. 

## 3. Pharmacokinetics Studies

Due to the rapid dispersion and removal, RA exhibits a low bioavailability and concentration in rat plasma. This may be due to the high molecular mass (897.1 D) of RA and poor membrane permeability as a result of the hydrophilic sugars in the RA structure. Moreover, this compound suffers from a short half-life and less systematic exposure due to the vulnerability of fast and large scale biliary excretion through active transport, as RA belongs to the saponins family [80]. A simple, fast and sensitive high-performance liquid chromatography electro spray ionization tandem mass spectrometry (LC-ESI-MS/MS) method was employed to characterize the presence of RA in rat plasma. In vivo studies were conducted using an RA concentration of 2 mg/kg administered orally and intravenously to S.D rats. With 2.04−6.52% precision, 70% of RA was recovered without any clear matrix effect. The analysis indicated that due to poor lipid solubility, RA depicted less systemic absorption and thereby less bioavailability of 0.295% in plasma samples. This is identical to nearly all saponins reported previously in literature [81].

Another study was conducted using 0.75 mg/kg intravenous and intraperitoneal administrations of RA in Sprague Dawley (S.D.) rats. The blood sample was investigated at different time intervals which extended over 24 h for RA and glycyrrhetinic acid (internal standard) and analyzed under negative electrospray ionization in multiple reaction monitoring (MRM) mode [28]. The study indicated that the absolute recovery of RA was greater than 90.3% with a retention time of 2.1 min. The apparent distribution volume of RA was 0.11 L/kg less than total body volume 0.67 L/kg reflecting that RA widely disperses in blood compartments as compared to extravascular tissues. A recent in vivo study was carried out on mice by an LC-MS/MS system [82]. All mice were fed a single oral dose of 1.5 mg/kg and a blood sample was taken at different time intervals. The plasma of samples was stored at −80 °C for analysis. The study reveals that maximum concentration of RA was absorbed quickly at time duration of 0.33 h. They found low bioavailability with highest concentration of 12.3 μg/L. This dose of RA was the same as found in previous studies. Furthermore, RA exhibited rapid removal with a half-life of 3.5 h and it couldn’t be detected in the plasma after 6h. The pharmacokinetic study of RA and its tissue distribution was observed in mice. After oral administration, among gastro-intestinal organs, the highest concentration of RA was detected in the stomach, followed by the colon and caecum. However, after 4 h of oral administration, RA could not be detected in any gastrointestinal organ [83]. The rapid dispersion and removal of RA was in accordance with the low bioavailability and low concentrations in rat plasma [28]. These studies depicted the low bioavailability of RA in plasma, although proper routes of administration are yet to be determined. 

## 4. Role of RA in Cancer Prevention and Treatment

The therapeutic potential of RA against various cancers, including prostate, breast, gastric, colon, hepatic, cholangiocarcinoma, osteosarcoma, and glioblastoma has been extensively reported. Studies indicated that RA triggers apoptosis, inhibits proliferation, angiogenesis, and proved to be anti-metastatic in different cancers (Table 1). Hence, the mechanism of action of RA against different malignancies are described below. 

### 4.1. Breast Cancer

Breast cancer is a clinically complicated condition with a high incidence and mortality rate in developed countries [84,85,86,87,88]. According to the vicious cycle of bone metastasis, bone cells can interact with breast cancer cells in which tumor cells release pro-osteoclastic factors to induce osteoclastogenesis, while the bone matrix releases pro-tumorigenic growth factors. This in turn can augment tumor expansion [89,90]. To impose osteoclast differentiation and consequently bone resorption, breast cancer cells release inflammatory cytokines such as nuclear factor-κB (NF-κB) ligand (RANKL), which leads to the activation of downstream signaling pathways such as MAPK, NF-κB, and SRC/AKT [70,71,72]. The studies of the therapeutic effect of RA specifically showed the inhibition of SRC/AKT pathway. In vitro, RA effectively prevented the RANKL associated osteoclastogenesis on bone marrow-derived macrophages (BMMs) and osteoblast differentiation [36]. Consistent with this result, treatment of RA at different concentrations considerably inhibited the Ti-particle-induced osteolysis by downregulating SRC/AKT signaling pathway in in vivo mice calvarial model. Hence, RA inhibited RANKL regulated SRC expression, and ultimately downregulated the AKT pathway which leads to the inhibition of osteoclastogenesis [36]. To get insight into the RA mechanism and the pathway, researchers found that RA treatment can cause inhibition of AKT phosphorylation, which was later rescued by using AKT activator SC-79. Hence these findings proved that RA specifically directed PI3K/Akt pathway, without interfering with MAPK and NF-κB signaling pathways. In vivo, Ra has been reported to restore osteolysis and decreased trabecular separation in the osteolysis female mouse model.

Another article reported by Guan et al., indicated that RA treatment induces cellular apoptosis and inhibits invasion and angiogenesis through the modulation of P13K/AKT/mTOR signaling pathway [64,65]. Importantly, this inhibition specifically reduced the multidrug resistance, which rendered various antitumor therapies as ineffective in breast cancer. Moreover, application of RA on breast cancer cell lines (MDA-MB-231, MCF-7 and T47D) induced autophagy and cytotoxicity through modulation of Akt-mTOR-eEF-2K signaling pathway, as evidenced by an elevated level of autophagy marker LC3, which is also considered as one of the hallmarks of cancer [67]. Upon pre-treatment with chloroquine at 20 μmol/L (an autophagy inhibitor), RA considerably enhanced cytotoxicity and induced apoptosis via intrinsic pathways through downregulating anti-apoptotic proteins (Bcl-2, Bcl-xL and Mcl-1) and upregulating caspase-3 and PARP expression. The morphological changes induced by apoptosis were also noticed by nuclear shrinkage, chromatin condensation, and fragmentation. Hence, it is suggested that through the modulation of AKT and autophagy, RA can reinforce the substantial level of apoptosis in breast cancer cells. 

### 4.2. Cholangiocarcinoma

Cholangiocarcinoma (CCA) is a rare biliary adenocarcinoma characterized by aggressive metastatic and invasive tumors with poor outcomes [91,92]. Due to late diagnosis and vigorous growth, effective treatment is not available against CCA yet. To date, surgical resection remains the only therapeutic approach, but most cases result in death due to late diagnosis [93]. In addition, the resistance of cancerous cells towards available chemotherapeutics is one of the leading challenges [94]. 5-Fluorouracil (5-FU) is currently used in clinics as a chemotherapeutic agent, but due to resistance against CCA cells, the efficacy of this compound has been remarkably reduced Antitumor effect of 5-FU is enhanced by rosemary extract in both drug sensitive and resistant colon cancer cells [94]. The administration of RA promoted apoptosis in four cholangiocarcinoma cell lines. Amidst all, RA specifically impaired migration and hindered colony formation in LIPF155C and RBE cell lines. RA through its antitumor effect, sensitized bile duct cancer cells toward 5-FU and further mediated apoptosis in a 5-FU-resistant cell line. The Wee1 belongs to a protein kinase family involved in cell proliferation by halting cell cycle arrest in tumor cells [95,96]. RA increased apoptosis and impaired cellular functions via the activation of the Wee1 dependent signaling mechanism in RBE cell lines. The combined treatment of RA along with 5-FU (35μM) synergistically downregulated the expression of Bcl-2, cyclooxygenase-2, and Wee1 and upregulated Bax. In addition, RA mediated cell cycle arrest by modulating cell cycle-related protein cyclin E/D1. Through this process, the cyclin family forms an orchestrated series of molecular complexes to regulate cell cycle progression. In response to DNA damage, cyclin D-Cdk4/6 and cyclin E-Cdk2 in particular regulates G1-S transition [95,97]. Therefore, a high level of E/D1 cyclin protein can act as pro-apoptotic factors, possibly involved in the sensitization of cancerous cells toward radiation [98]. Hence, the synergistic effect of RA and 5-FU could be a potential therapeutic approach for cholangiocarcinoma.

### 4.3. Colorectal Cancer

Colorectal cancer (CRC) is a highly prevalent cancer globally and the third-leading cause of death after lung and breast cancer [99,100]. Cancer cells have the ability to induce angiogenesis, invasion, and metastasis to invade other parts of the body [101]. Ying and co-workers highlighted in their research that RA inhibited the progression of angiogenesis and metastasis of colorectal tumor [79]. RA successfully inhibited HUVEC proliferation and orchestrated the process of angiogenesis, including endothelial cell proliferation, motility, and capillary-like tube formation without affecting HCT-15 healthy endothelial cells, specifying its activity only against tumor endothelial cell. In the chick embryo chorioallantoic membrane (CAM) model, RA treatment effectively blocked blood vessel formation in a dose-dependent manner. The study was further extended to zebrafish model, where RA treatment was shown to disrupt nearly 68% of intersegmental vessel (ISVs) formation along with the deformed morphology of zebrafish. In HCT-15 xenograft mice models, RA dose-dependently showed substantial antiangiogenic potential through a remarkable decrease in micro vessel density (MVD) along with reduced tumor growth and weight. The above mentioned antiangiogenic effect of RA was mediated through the phosphorylation of VEGF-induced vascular endothelial growth factor 2 and the inhibition of downstream kinases and signaling pathways including focal adhesion kinase (FAK), JAK2, PLCγ1, Src, and Akt inhibition, involved in survival, migration and proliferation of EC. This mechanism was further hypothesized by molecular docking simulation, based on which RA pentacyclic triterpene moiety docked at the ATP-binding pocket of VEGFR2 kinase domain occupied with six amino acids and facilitate the formation of VEGFR2-RA complex to inhibit downstream molecular pathways.

PI3K/Akt signaling pathway constitutively expressed in malignant cells and activate several downstream proteins to regulate cell proliferation, cell metabolism, cell survival, and angiogenesis [62,63,64,65]. Activated PI3K/Akt pathway indirectly activates mTOR pathway which subsequently triggers activation of genes involved in apoptosis and cell cycle progression [8]. Chunqin et al. discovered that RA treatment dose-dependently stimulated apoptosis, G0/G1 cell cycle arrest and blocked cell cycle proliferation in colorectal HCT-116 cell line possibly through inhibiting the PI3K/AKT signaling pathway [102]. In the HCT116-xenograft mouse model, RA significantly reduced the tumor growth, whereas apoptotic cells were also seen in tumor tissue [102]. RA treatment significantly decreased the protein level of cyclin D1, cyclin E, p-PI3K, and p-AKT, suggested the strong anti-tumor potential of RA in vivo HCT116 cells induced xenograft mice.

Yu and co-workers discovered the anti-proliferative and apoptotic effects of RA in colorectal LOVO and SW480 cell lines [103]. The study was further extended to in vivo xenograft mouse model, where RA had significantly inhibited the tumor growth through modulation of Wnt/β-catenin signaling via downregulation of p-LRP6, upregulation of AKT inactivation, inhibition of β-catenin and removal of GSK-3β inhibition. In addition, RA prevented tumor through modulation of NF-κB signaling pathway, inhibited phosphorylation of IκB-α which led to an induction of the mitochondrial apoptotic pathway. 

### 4.4. Glioblastoma

Glioblastoma multiforme (GBM) is an incurable primary brain tumor with a low long-term survival rate in affected patients. According to a recent report, the estimated incidence rate of GBM is around 5.62 per 100,000 people and the rate is growing [104,105]. Despite standard treatment, the aberrant metastatic potential of brain tumor cells, and incomplete surgical resection has declined the survival rate of less than one year after diagnosis [106]. Peng and coworkers have suggested the therapeutic potential of RA in reducing cell viability of four GBM cell lines (G112, T98, U87, and U251) compared with control cells [107]. RA treatment in these cell lines effectively reduced the level of MMP-2 and MMP-9, linked to the invasive and migratory potential of cancer cells. Treatment with RA induced apoptosis in glioma cells through increased ROS production, which lead to the activation of Jun N-terminal kinase (JNK) signaling pathway, which mediated high Bax/Bcl-2 ratio and subsequently caspase-3 and PARP upregulation. The activation of ROS/JNK signaling pathway was further verified by targeting cells with antioxidant NAC (N-Acetyl-L-cysteine) and caspase inhibitor (z-VAD-fmk), as a result reduced apoptotic rate, p-JNK and caspase-3 level authenticated the activation of this pathway in colorectal cells (T98 and U251). Interestingly, appearance of apoptotic cells after z-VAD-fmk treatment highlighted the occurrence of other possible mechanism in inducing apoptosis. Therefore, RA induced cell death also triggered through another mechanism along with apoptotic pathway. Furthermore, treatment of RA induced autophagy in glioma cells, since it is known to either support or inhibit apoptotic signaling. However, in this study, the application of autophagy inhibitors class III PI3K and 3-MA, exacerbated the RA mediated apoptosis in U251 glioma cell as noticed with caspase-3 overexpression. In U251-harbouring xenografts nude mice model, RA treatment exhibited curative effect with a significant drop in tumor size and induced apoptosis as noted by elevated level of caspase-3, LC3-I to LC3-II conversion and p-JNK.

### 4.5. Gastric Cancer

Gastric cancer (GC) is the third most extensively frequent cancer in males due to the increased resistance of gastric cancer cells toward clinically used chemotherapeutic agents [3,105,108]. The gastric cancer patient typically suffers relapse after surgery, which reduces the survival rate to less than five years [109,110,111,112,113,114]. Gang et al. discovered that RA treatment activated apoptosis and invasion in three dissimilar differentiation stage gastric cancer (GC) cells (BGC-823, MKN-28, and SGC-7901) [115]. Amongst all, RA remarkably reduced proliferation, adhesion, invasion, and migration in BGC-823 cells. However, a study reported by Hao et al., [37] also suggested the anti-proliferative potential of RA against SGC-7901 cells in a concentration dependent manner. Administration of RA on these cells hinder proliferation and induced apoptosis via mitochondrial apoptotic signaling cascade led to a drop in Bcl-2, Bcl-xL, survivin expression, whereas simultaneously upregulated the pro-apoptotic Bax, caspase-3, caspase-8, caspase-9 expression in addition to activation of PARP cleavage. Besides, the RA also attenuated the invasive, migratory, and angiogenic potential of tumor cells by inhibiting MMP-2, MMP-9, MMP-14, and Roc proteins. On the contrary, the E-cadherin (E-cad) expression and reversion inducing cysteine-rich protein with Kazal motifs (RECK) was significantly upregulated, which negatively associated with MMPs, therefore supported the notion that RA specifically prevented angiogenesis via MMPs inhibition. Furthermore, treatment of RA induced apoptosis and autophagy through modulation of p38/MAPK pathway as indicated by high level of p-p38 and ERK level in GC cells [75,76]. Moreover, LC3I to LC3II conversion along with phosphorylation of p-mTOR notably depicted the existence of autophagy in these cells, which probably protect cancerous cells from apoptosis and reduce the inhibitory potential of target compound [115]. Therefore, employing an autophagy inhibitor could be the best choice in RA-mediated apoptosis against gastric cancer cells.

### 4.6. Hepatocellular Carcinoma (HCC)

Hepatocellular carcinoma (HCC) is the aggressive malignancy of the liver and the second leading cause of mortality in the world [3]. Cisplatin is a well-established alkylating compound used regularly in human hepatocellular carcinoma (HCC) chemotherapies and radiotherapy [116], though accompanied by remarkable cytotoxicity [117,118]. Clinically, it is used in combination with other drugs to reduce toxicity. Cisplatin performs its function by repressing the tumor cellular DNA repair process [119]. RA, coupled with cisplatin, reduced its toxicity and showed a remarkable synergistic effect against tumor on QGY-7703 cells based on combination index (CI) values less than 0.8. Anaerobic condition in the tumor microenvironment enhanced tumor cell growth and metastasis due to which intracellular ROS production is used to evaluate the underlying proliferation or metastasis of HCCs [120]. RA, in combination with cisplatin, increases the ROS level and also facilitates the oxygen metabolism in HCC cells, including HepG2 and SMMC-7721. In addition, RA enhanced cisplatin effect through increasing sensitivity of resistant cells, which further activated their apoptosis. RA significantly inhibited proliferation by inducing S phase cell cycle arrest, whereas cisplatin induced cell cycle arrest at the G0/G1 stage [121]. The mRNA expression level of apoptotic genes was upregulated for p53 and Bax, whereas simultaneously led to a reduction of Bcl-2 and Survivin proteins [38]. Hence, RA treatment together with cisplatin, could serve as a potential therapeutic target in reducing toxicity of cancerous cells toward commercially available chemotherapeutic agent.

### 4.7. Lung Cancer

Lung cancer is the heterogeneous disease of lungs and the leading cause of global cancer mortality [3]. Among all lung cancers, 80% of cases belong to non-small cell lung cancer (NSCLC) with gradual increasing incidence and mortality rate [122]. Treatment of RA remarkably inhibited cell proliferation and blocked cell cycle progression of NSCLC H460 cells. RA significantly reduced proliferation of H460 in a concentration-dependent manner. RA modulated the Akt mediated G2/M phase arrest, down-regulated Bcl-2 and cleaved PARP expression, leading to reduced H460 cell proliferation apoptosis induction. These findings suggested the strong therapeutic potential of RA in combating cancer cells and increasing the survival of patients affected by NSCLC [35].

### 4.8. Osteosarcoma

Osteosarcoma is a highly aggressive bone tumor, associated with poor survival. Treatment with RA was shown to exhibit an anti-tumor effect via JNK mediated mitochondrial apoptosis pathway and inhibited metastasis on human osteosarcoma cell lines [123]. RA downregulated Bcl-2/Bax ratio, upregulated cleaved caspase-3 and PARP, as crucial components of apoptosis. RA increased cell apoptotic activity by ROS/JNK phosphorylation and downregulation of NF-κB transcriptional activity through a low level of p-IκBα and p65 [123]. After pre-treatment with RA, inhibition of p65 results in the sensitization of osteosarcoma cells. In addition, RA represses invasion and migration by downregulating MMP-2/9 level mediated with NF-κB pathway [123]. An in vivo study following exposure of RA in ROS xenograft models also confirmed the anti-cancer effects of RA on human osteosarcoma. Zhuoying and coworkers [124] suggested that RA treatment induced apoptosis and abrogated proliferation in osteosarcoma cells through modulation of the JNK/c-Jun and STAT3 signaling pathways. An in vivo study on tibial xenograft tumor model, administration of apoptosis in OS cells, which lead to the reduction of tumor size. Furthermore, another study indicated that RA had shown an antitumor effect in both drug-resistant and non-resistant OS cells [78]. Treatment with RA mediated apoptosis, blocked cell proliferation, as well as restricted colony formation by mediating interleukin-6 (IL-6) induced JAK2/STAT3 pathway. By contrast, RA treatment was shown to increase the levels of MDR1 and STAT3 in resistant OS cells, whereas high expression of these protein is related to the chemoresistance. The expression of MDR1 protein in both drug-sensitive and resistant OS cells, along with downregulation of STAT3 by using siRNA, increased the sensitivity of these cells toward doxorubicin treatment. The combine treatment of RA and doxorubicin increased the doxorubicin uptake by cell, result in increasing toxicity, ablated efflux, and reduced MDR1 expression in drug resistant cells by modulating STAT3 phosphorylation. Consistent with these results, RA reduced tumor growth by promoting apoptosis in doxorubicin-resistant OS tibia orthotopic model. Therefore, RA serves as a potential therapeutic for doxorubicin resistance treatment in OS.

### 4.9. Prostate Cancer

In men, prostate cancer is the most common type of malignancy and does not have therapeutic options in the advanced state [125,126]. The castration resistance after androgen deprivation therapy is the ultimate cause of death in castration-resistant prostate cancer (CRPC). The upregulation of full-length androgen receptor (AR-FL) and splice variants (AR-Vs, AR-V7, ARv567es, and AR-V9) are most likely involved in poor prognosis and castration resistance. It is, therefore, an urgent need to develop a drug that acts as a reactivation mechanism in combating CRPC. A recent report showed that RA has effectively attenuated the transcription of AR-FL and splice variants AR-Vs. Docetaxel is a first-line chemotherapy drug that has been implicated in CRPC and mediates its effect through stabilizing microtubules; however, AR-V7 is localized in the nucleus and is independent of the microtubule. Therefore, it remains insensitive toward docetaxel inhibition [127,128]. In CRPC cells, RA was reported to selectively target both the full-length AR-FL and splice variant AR-V mRNA expression and increase the growth inhibitory efficacy of docetaxel synergistically in both time- and dose dependent manners. RA inhibition is entirely dependent on AR other than androgen, as AR-null cells are left unaffected by RA treatment. This inhibition is of utmost importance because none of the drugs available in the market can directly target the full length and splice variants of the AR. However, compounds other than RA have been shown to decrease the levels of AR-FL and AR-Vs pre-clinically [129,130,131]. Taken together, the study provided a rationale for RA and its combination treatment against CRPC.

### 4.10. Chemosensitizing Properties of RA

Surgery, radiotherapy and chemotherapy are conventional clinical treatments for cancer [132]. However, more and more tumors have become resistant to chemotherapy in recent years, which has become a major obstacle to cancer treatment [111,113,114,133,134,135]. 5-FU is the most common chemotherapeutic compound for cancer treatment [69]. RA sensitized cholangiocarcinoma cell lines to 5-FU treatment and ameliorated 5-FU resistance in bile duct cancer cells through activating multiple cell cycle and apoptosis-related factors, such as COX-2, Bax, Bcl-2, and cyclins E/D1 [136]. RA may also have the potential to enhance the growth inhibitory efficacy of docetaxel, the first-line chemotherapy for prostate cancer [137]. The growth and survival of prostate cancer cells rely on androgen receptor (AR) [136]. Splice variants of AR (AR-V) expression have been proposed to be a mechanism of docetaxel resistance. RA enhanced the growth inhibitory efficacy of docetaxel through suppressing both full-length (AR-FL) and AR-V expression and activities. Peng *et al.,* reported that the activation of STAT3/NFIL3 signaling axis results chemotherapeutic resistance. In addition, RA reversed STAT3/NFIL3 signaling axis-mediated chemotherapy resistance in drug-resistance choriocarcinoma cell lines such as JEG-3/MTX (methotrexate-resistant-JEG-3 cells), JEG-3/5-FU-resistant-JEG-3 cells), and JEG-3/VP16 (etoposide-resistant-JEG-3 cells) [138].

## 5. Limitations and Future Prospects

The diverse pharmacological effects of RA have been analyzed in this review, indicating the therapeutic potential of RA against numerous cancer cell lines. Evidence has suggested that RA shows anticancer potential both in vitro and in vivo animal models. However, in vivo studies are confined to some cancers such as breast, colorectal, prostate and osteosarcoma. In addition, based on previous pharmacokinetic data, low bioavailability of RA in the systemic circulation is a major concern, therefore, there is a need to explore in depth mechanisms in order to increase the compound’s bioavailability and to retain the metabolites for an optimal effect. Furthermore, despite having a large number of combination studies with other chemotherapeutic drugs, no clinical study is reported as yet. Hence, in an attempt to get further insights, available data can be employed in clinical settings.

## 6. Conclusions

This review provides a comprehensive detail about the diverse anticancer potential of RA in both in vitro and in vivo studies. The effect of RA is mainly exerted through the induction of apoptosis, cell cycle arrest and the inhibition of cell proliferation along with modulating cell signaling mechanisms in breast, cholangiocarcinoma, colorectal, liver, lung, prostate and osteosarcoma. Amidst various signaling pathways, PI3K/AKT has been the most significantly modulated by RA in different cancers. Furthermore, RA has induced synergistic effects in combination with other chemotherapeutic drug and increases sensitivity of tumor cells to apoptosis without posing toxic effect. Therefore, RA can be used as a novel anticancer agent against those malignancies that have developed resistance to chemotherapy. In preclinical studies, RA significantly reduced tumor growth, tumor size and metastasis. However, the effective concentrations against tumor cells varies depending on the type of cell and in vivo model system. Hence, clinical trials are required to establish the effectiveness of RA in clinical settings. The detailed study concluded that RA can be used as a promising anticancer compound.

## Figures and Tables

**Table 1:** A list of different cancers affected by RA under in vitro and in vivo settings.

Cancer	Model	Experiment	Dosage	Anti-Cancer	Mechanisms of Action	Ref
Colorectal cancer	SW480, LOVO, Caco-2, HT-29	In vitro	0–50 mM, 0.4 μM	Apoptosis, Anti-proliferative, Anti-angiogenic	↓Bax/Bcl-2, ↓Wnt/β-catenin, ↓AKT, ↓GSK-3β, ↓LRP6-p, ↓c-Myc, ↓cyclin D1, ↓NF-kB, ↑cell cycle arrest	[103]
	HCT-15 xenograft mice	In vivo	3 μM	Anti-proliferative, Anti-invasive, Anti-migratory, Anti-angiogenic	↓VEGF, ↓VEGFR2, ↓PLCγ1, ↓JAK2, ↓FAK, ↓Src, ↓Akt	[79]
	HCT116 HCT116-xenograft mouse	In vitro In vivo	1–16 mM	Apoptosis, Reduce tumor growth	↑caspase-3, ↑PARP, ↑BAX, ↓p-PI3K, ↓p-AKT, ↓cyclin D1, ↓cyclin E, ↓CDK4, ↓CDK2, ↑G0/G1 phase arrest	[102]
Breast cancer	BMMs, MDA-MB-231 MCF-7, T47D BALB/c nu/nu Female mice	In vitro In vivo	0–0.8 mM, 2–8 μmol/L, 2–10 μmol/L	Apoptosis, Anti-invasive, Anti-proliferative, Reverse Osteolysis	↓MAPK, ↓NF-κB, ↓SRC/AKT ↓AKT/mTOR signaling ↑Akt-mTOR-eEF-2K	[36], [67]
Cholangiocarcinoma	RBE, LIPF155C, LIPF178C, LICCF	In vitro	0–160 μg/mL, 13 μg/mL 13 μg/mL + 50 μmol/L RBE/5-FU	Apoptosis, Anti-migratory, Inhibit colony formation	↓Cox-2, ↓Bcl-2, ↓Wee1, ↑Bax, ↑cyclin D1, ↑cyclin E	[69]
Glioblastoma	G112, T98, U251, U87 U251-harboring xenografts in nude mice	In vitro In vivo	0–8 mM, 4 μM RA 1 mg/kg, 2 mg/kg bw	Apoptosis, Anti-proliferative Reduce Tumor size, body weight	↑ROS/JNK, ↑Bax, ↓Bcl-2, ↑caspase-3, ↑PARP	[107]
Hepatocellular carcinoma	QGY-770	In vitro	0–55.8 μM 29.22 mM 14.71 μM RA + 4.92 μM Cisplatin	Reduce ROS	↑G0/G1 phase arrest ↓p53, ↑Bax, ↓Bcl-2, ↓survivin	[38]
Prostate cancer	22Rv1, DU145, PC-3	In vitro	0–6 μmol/L plasma conc. 4.5 μmol/L, 3 μmol/L	Inhibit growth,	↓AR-FL, ↓AR-V7	[136]
Gastric cancer	SGC-7901 BGC-823, MKN-28, SGC-7901	In vitro	0–16 μM, 8 mM 8 µM, 16 mL + 50 µg/mL 5-FU	Apoptosis, Anti-proliferative, Anti-invasive, Anti-migratory, Reduce autophagy	↑Bax, ↑caspase-3, ↑caspase-8, ↑caspase-9, ↑PARP, ↓Bcl-2, ↓Bcl-xL, ↓Beclin-1, ↓Survivin, ↑RECK, ↑E-cad, ↑MMP-2, ↑MMP-9, ↑MMP-14, ↑Rhoc ↑ATG5, ↑ATG7, ↑p-p38, ↑ERK, ↑mTOR, ↓IP3 level	[37] [115]
Osteosarcoma	MG-63, HOS HOS cell xenograft mice tibial xenograft tumor mice CCK8 orthotopic Chemoresistant OS mice	In vitro In vivo	0.2-50 μM 1.25, 2.5, 5 mg/kg 5 mg/kg + 1 mg/kg Dox	Apoptosis, Anti-invasive, Anti-metastatic, Anti-proliferative, inhibit osteosarcoma, Reduce tumor growth	↓Bcl-2, ↑Bax ratio, ↑caspase-3, ↑PARP, ↓NF-κB, ↓MMP-2, ↓MMP-9 ↑ROS/JNK, ↑ERK1/2 ↑caspase-3, ↑caspase-8, ↑caspase-9, ↑PARP ↑JAK2/STAT3, ↑MDR1, ↑STAT3	[123] [78] [124]

## Abbreviations

5-FU	5-Fluorouracil
AR-FL	Androgen Receptor Full Length
ARVS	Androgen Receptor Splice Variant
BAX	Bcl2-Associated X Protein
BCL-2	B-Cell Lymphoma-2
BCL-XL	B-Cell Lymphoma-Extra Large
BMM	Bone Marrow-Derived Macrophages
CDK1	Cyclin Dependent Kinase 1
CDK2	Cyclin Dependent Kinase 2
CDK4	Cyclin Dependent Kinase 4
CDK6	Cyclin Dependent Kinase 6
CI	Combination Index
CRC	Colorectal Cancer
CRPC	Castration Resistance Prostate Cancer
EGFR	Epidermal Cell Growth Factor Receptor
EMT	Epithelial-Mesenchymal Transition
ERK	Extracellular Signal Regulated Kinase
FAK	Focal Adhesion Kinase
FOXM1	Forkhead Box M1
FOXO3A	Forkhead Box Class O 3a
GBM	Glioblastoma Multiforme
HCC	Hepatocellular Carcinoma
HDAC	Histone Deacetylases
IFGR	Insulin-Like Growth Factors
IL-6	Interleukin 6
IL-B	Interleukin B
JNK	c-Jun N-Terminal Kinase
LC–ESI-MS/MS	High-Performance Liquid Chromatography Electrospray Ionization Tandem Mass Spectrometry
LRP6	LDL Receptor-Related Protein 6
MAPK	Mitogen-Activated Protein Kinases
MMP	Matrix Metalloproteinase
MMP-1	Matrix Metalloproteinase-1
MMP-2	Matrix Metalloproteinase-2
MMP-9	Matrix Metalloproteinase-9
MRM	Multiple Reaction Monitoring
MVD	Micro Vessel Density
NAC	Acetylcysteine
NF-kB	Nuclear Factor Kappa Light Chain Enhancer of Activated B Cells
OS	Osteosarcoma
PARP	Poly ADP-Ribose Polymerase
PDK-1	Phosphoinositide Dependent Kinase-1
PI3K	Phosphatidylinositol 3-Kinase
PIP_3_	Phosphatidylinositol Trisphosphate
PKB	Protein Kinase B
RA	Raddeanin A
RANKL	Inflammatory Cytokines Like Nuclear Factor-ΚB (NF-kB) Ligand
RECK	Cysteine-Rich Protein with Kazal Motifs
ROS	Reactive Oxygen Species
RTK	Receptor Tyrosine Kinases
S.D	Sprague Dawley
STAT3	Signal Transducer and Activator Of Transcription 3
TIMP2	Timp Metallopeptidase Inhibitor 2
TRAF6	TNF Receptor-Associated Factor 6
VEGFA	Vascular Endothelial Growth Factor A
XIAP	X-Linked Inhibitor of Apoptosis Protein
